# Robot-Assisted Therapy in Upper Extremity Hemiparesis: Overview of an Evidence-Based Approach

**DOI:** 10.3389/fneur.2019.00412

**Published:** 2019-04-24

**Authors:** Christophe Duret, Anne-Gaëlle Grosmaire, Hermano Igo Krebs

**Affiliations:** ^1^Centre de Rééducation Fonctionnelle Les Trois Soleils, Médecine Physique et de Réadaptation, Unité de Neurorééducation, Boissise-Le-Roi, France; ^2^Centre Hospitalier Sud Francilien, Neurologie, Corbeil-Essonnes, France; ^3^Department of Mechanical Engineering, Massachusetts Institute of Technology, Cambridge, MA, United States; ^4^Department of Neurology, University of Maryland, Baltimore, MD, United States; ^5^Department of Physical Medicine and Rehabilitation, Fujita Health University, Nagoya, Japan; ^6^Institute of Neuroscience, University of Newcastle, Newcastle upon Tyne, United Kingdom,; ^7^Department of Mechanical Sciences and Bioengineering, Osaka University, Osaka, Japan; ^8^Wolfson School of Mechanical, Electrical, and Manufacturing Engineering, Loughborough University, Loughborough, United Kingdom

**Keywords:** hemiparesis, rehabilitation robotics, robot-assisted therapy, upper extremity, stroke

## Abstract

Robot-mediated therapy is an innovative form of rehabilitation that enables highly repetitive, intensive, adaptive, and quantifiable physical training. It has been increasingly used to restore loss of motor function, mainly in stroke survivors suffering from an upper limb paresis. Multiple studies collated in a growing number of review articles showed the positive effects on motor impairment, less clearly on functional limitations. After describing the current status of robotic therapy after upper limb paresis due to stroke, this overview addresses basic principles related to robotic therapy applied to upper limb paresis. We demonstrate how this innovation is an evidence-based approach in that it meets both the improved clinical and more fundamental knowledge-base about regaining effective motor function after stroke and the need of more objective, flexible and controlled therapeutic paradigms.

## Introduction

Robot-mediated rehabilitation is an innovative exercise-based therapy using robotic devices that enable the implementation of highly repetitive, intensive, adaptive, and quantifiable physical training. Since the first clinical studies with the MIT-Manus robot (1), robotic applications have been increasingly used to restore loss of motor function, mainly in stroke survivors suffering from an upper limb paresis but also in cerebral palsy (2), multiple sclerosis (3), spinal cord injury (4), and other disease types. Thus, multiple studies suggested that robot-assisted training, integrated into a multidisciplinary program, resulted in an additional reduction of motor impairments in comparison to usual care alone in different stages of stroke recovery: namely, acute (5–7), subacute (1, 8), and chronic phases after the stroke onset (9–11). Typically, patients engaged in the robotic therapy showed an impairment reduction of 5 points or more in the Fugl-Meyer assessment as compared to usual care. Of notice, rehabilitation studies conducted during the chronic stroke phase suggest that a 5-point differential represents the minimum clinically important difference (MCID), i.e., the magnitude of change that is necessary to produce real-world benefits for patients (12). These results were collated in multiple review articles and meta-analyses (13–17). In contrast, the advantage of robotic training over usual care in terms of functional benefit is less clear, but there are recent results that suggest how best to organize training to achieve superior results in terms of both impairment and function (18). Indeed, the use of the robotic tool has allowed us the parse and study the ingredients that should form an efficacious and efficient rehabilitation program. The aim of this paper is to provide a general overview of the current state of robotic training in upper limb rehabilitation after stroke, to analyze the rationale behind its use, and to discuss our working model on how to more effectively employ robotics to promote motor recovery after stroke.

## Upper Extremity Robotic Therapy: Current Status

Robotic systems used in the field of neurorehabilitation can be organized under two basic categories: exoskeleton and end-effector type robots. Exoskeleton robotic systems allow us to accurately determine the kinematic configuration of human joints, while end-effector type robots exert forces only in the most distal part of the affected limb. A growing number of commercial robotic devices have been developed employing either configuration. Examples of exoskeleton type include the Armeo®Spring, Armeo®Power, and Myomo® and of end-effector type include the InMotion™, Burt®, Kinarm™ and REAplan®. Both categories enable the implementation of intensive training and there are many other devices in different stages of development or commercialization (19, 20).

The last decade has seen an exponential growth in both the number of devices as well as clinical trials. The results coalesced in a set of systematic reviews, meta-analyses (13–17) and guidelines such as those published by the American Heart Association and the Veterans Administration (AHA and VA) (21). There is a clear consensus that upper limb therapy using robotic devices over 30–60-min sessions, is safe despite the larger number of movement repetitions (14).

This technic is feasible and showed a high rate of eligibility; in the VA ROBOTICS (9, 11) study, nearly two thirds of interviewed stroke survivors were enrolled in the study. As a comparison the EXCITE cohort of constraint-induced movement therapy enrolled only 6% of the screened patients participated (22). On that issue, it is relevant to notice the admission criteria of both chronic stroke studies. ROBOTICS enrolled subjects with Fugl-Meyer assessment (FMA) of 38 or lower (out of 66) while EXCITE typically enrolled subjects with an FMA of 42 or higher. Duret and colleagues demonstrated that the target population, based on motor impairments, seems to be broader in the robotic intervention which includes patients with severe motor impairments, a group that typically has not seen much benefit from usual care (23). Indeed, Duret found that more severely impaired patients benefited more from robot-assisted training and that co-factors such as age, aphasia, and neglect had no impact on the amount of repetitive movements performed and were not contraindicated. Furthermore, all patients enrolled in robotic training were satisfied with the intervention. This result is consistent with the literature (24).

The main outcome result is that robotic therapy led to significantly more improvement in impairment as compared to conventional usual care, but only slightly more on motor function of the limb segments targeted by the robotic device (16). For example, Bertani et al. (15) and Zhang et al. (17) found that robotic training was more effective in reducing motor impairment than conventional usual care therapy in patients with chronic stroke, and further meta-analyses suggested that using robotic therapy as an adjunct to conventional usual care treatment is more effective than robotic training alone (13–17). Other examples of disproven beliefs: many rehabilitation professionals mistakenly expected significant increase of muscle hyperactivity and shoulder pain due to the intensive training. Most studies showed just the opposite, i.e., that intensive robotic training was associated with tone reduction as compared to the usual care groups (9, 25, 26). These results are shattering the resistance to the widespread adoption of robotic therapy as a therapeutic modality post-stroke.

That said, not all is rosy. Superior changes in functional outcomes were more controversial until the very last years as most studies and reviews concluded that robotic therapy did not improve activities of daily living beyond traditional care. One first step was reached in 2015 with Mehrholz et al. (14), who found that robotic therapy can provide more functional benefits when compared to other interventions however with a quality of evidence low to very low. 2018 may have seen a decisive step in favor of robotic as the latest meta-analysis conducted by Mehrholz et al. (27) concluded that robot-assisted arm training may improve activities of daily living in the acute phase after stroke with a high quality of evidence However, the results must be interpreted with caution because of the high variability in trial designs as evidenced by the multicenter study (28) in which robotic rehabilitation using the Armeo®Spring, a non-motorized device, was compared to self-management with negative results on motor impairments and potential functional benefits in the robotic group.

The Robot Assisted Training for the Upper Limb after Stroke (RATULS) study (29) might clarify things and put everyone in agreement on the topic. Of notice, RATULS goes beyond the Veterans Administration ROBOTICS with chronic stroke or the French REM_AVC study with subacute stroke. RATULS included 770 stroke patients and covered all stroke phases, from acute to chronic, and it included a positive meaningful control in addition to usual care.

## Robotic as a Vehicle to Translate Intensity Into a Stroke Rehabilitation Program

Intensity is a key ingredient in an effective post-stroke motor rehabilitation program. Numerous clinical studies (30–33) demonstrated that significant changes in motor performance result from intensive training; these authors defined intensity as duration or number of sessions and postulated that any program should contain at least 16 h of exercise-based interventions to induce significant effects on activities of daily living, particularly in sub-acute patients (32).The concept of intensity as characterized by duration has been disputed by Page et al. (34). While the authors listed above and in most published studies within the last decade defined intensity mainly in terms of time and/or duration patients spent in therapy, Page and colleagues advocated to define “intensity” as the amount of work expended by the patients as they are performing a motor task and during a defined period of time. Although it is difficult to quantify this definition of intensity in usual care, it can be readily obtained in robot-mediated training considering not only the number of repetitions but also the assistance provided to the patient, hence characterizing active participation. In a retrospective study, Grosmaire and colleagues characterized upper limb motor activity during robotic training sessions in 16 patients with severe stroke by measuring patient participation within and across training sessions that consisted of hundreds of actively-assisted movements (35). They learned that, despite the large number of movements carried out during each session (at least 640) using an “assist-as-needed” robotic algorithm, the patients' active participation did not dwindle over the course of the same session as the robot reduced the assistance, thereby demanding more active patient participation. Moreover, the robotic assistance decreased from session to session, indicating a further increase in active participation over sessions. These results indicated that assist-as-needed-based upper limb robotic training promoted patient participation and can be associated with the number of repetitions during training (34). This result is in line with studies that have demonstrated that the number of repetitions is critical to alter neuronal structure and motor function after brain damage (36, 37). In non-primate animals following an induced stroke to the forepaw area, studies demonstrated that synaptogenesis and changes of cortical representations required a minimal number of repeated reaching tasks (38, 39). In human studies, plastic changes were demonstrated only after large numbers of movements of the upper extremity exceeding 300 repetitions per day (40). It is worth restating it: changes did not occur for a small number of repetitions, lower than 100 active movements per session (41). Current usual practices are not consistent with the evidence. Lang et al. (42) has shown that in a typical upper extremity rehabilitation session during the sub-acute post-stroke phase, there are <32 active movement attempts. Volpe et al. (43), Birkenmeier et al. (40), and Waddell et al. (44), have shown that chronic stroke survivors at all impairment levels were able to perform a large number of repetitive task-specific movements (at least 300) during a 1-hour therapy session. However, patients (and therapists) pointed out that they were exhausted at the end of the session and that they were not likely to work hard in the subsequent hours, which precludes patient participation in a comprehensive multidisciplinary program and therapists working effectively over the typical 8-hour working load.

Robot-mediated therapy has been an innovative approach to address the need of repetitive rehabilitation regimens. In sixteen robot-assisted, 45-minute sessions [4-days per week] added to the conventional upper limb therapy, Duret and colleagues found that severely impaired sub-acute patients performed an average of 734 movements per session (23). Moreover the number of reaching movements accomplished per session increased from 590 to 871 movements between the 1st and the 16th session. Pila et al. (45) also found similar results with the number of movements achieved by the patients ranging from 353 to 1295 per session. That said, while Volpe et al. (5) and the larger Veterans Administration (VA) RCT (9) demonstrated that robot-assisted training was superior to usual care, these studies showed parity between robotic and intensive comparison therapy (ICT) in chronic stroke. Health economics considerations in a context of budgetary constraint have resolved that the match demonstrated, at the least in the VA system, robot-mediated therapy is more efficient than ICT (46).

It is important to note that while intensity appears to require a minimum number of repetitions, it also appears to have a ceiling effect with only limited evidence supporting a monotonic dose-response relationship between intensity and upper limb motor function after stroke once we pass the minimum threshold (47). This lack of evidence was particularly highlighted by negative results in two recent studies using intensive programs (48, 49). Of notice, there are some caveats in that the first study did not provide details regarding the number of movements achieved in the “high dose” training groups (45) and the second study employed <300 movement attempts per session (49).

The robotic devices enable an easy quantification of the dose administered within a training session. The use of some robots demonstrated that the higher dose of robot-assisted training improved the motor outcomes when compared to a lower dose; Hsieh et al. (50) designed a clinical trial with 2 doses (number of repetitions) of robot-mediated rehabilitation in chronic stroke patients and showed that the higher dose resulted in the better motor outcomes. Burgar et al. (51) also found a similar relationship between the time spent in intervention of robotic therapy and the reduction of impairments in sub-acute stroke survivors. Indeed, one of the few studies that failed to demonstrate any advantage on the use of robotic for the upper extremity post-stroke as compared to usual care matched the number of robotic movements to the low number of movement attempts in usual care, in this case 60 movement attempts per hour session (52).

## Robotic Devices to Explore the Effectiveness of the Type of Assistance

There are a few small studies that have investigated the different types of robotic physical intervention. However, we should stress that most studies used robotic systems mainly in an active-assisted mode, utilizing mostly assist-as-needed paradigm (53). Lynch and colleagues demonstrated that a high intensity, continuous passive motion machine does not confer any advantage in terms of recovery of motor function over low intensity usual care in sub-acute (25). To be sure, attention is a critical component of the Hebbian model that posits that the patient must be actively attempting to perform the movement so that sensory feedback of the actual movement execution with robotic assistance promotes synaptogenesis or reinforcement of weakened or dormant pathways. In this regard, Lynch and colleagues work further alleviated concerns that high intensity robotic training might exacerbate tone or shoulder-hand syndrome. As mentioned earlier, many rehabilitation professionals were afraid that robotic therapy would lead to a significant increase of muscle hyperactivity and shoulder pain due to the intensive training. Actually most studies showed just the opposite (9, 25, 26). Hence, while inexpensive continuous passive motion machines might not lead to impairment reduction, they prevent secondary complications and might be included in the therapists' toolbox.

Another example examining the type of robotic intervention includes the work of Kahn et al. (52), who demonstrated that robot-assisted training could bring about similar outcomes compared to the same program performed without assistance. Notwithstanding these authors showed a better improvement in movement smoothness in the active non-assisted group. Fasoli et al. (54) and Stein et al. (55) also showed no difference on impairment reduction between patients carrying out an assist-as-needed rhythmic training and progressive resistance training.

Some studies compared controllers imposing force fields that either reduced or augmented the errors in trajectory. Force fields that augment errors open interesting perspectives for motor rehabilitation. For instance, Patton et al. (56) and Abdollahi et al. (57) have shown that the adaptation was better for fields that augmented the errors.

Reinkensmeyer et al. (58) emphasized an interest in having studies that would compare the effects of specific therapy based on the forces applied on the impaired limb to determine the optimal exercise regimen. Searching the level of assistance or type of optimal interaction required to stimulate motor recovery seems possible with robotic devices. Wolbrecht et al. (59) demonstrated the value of adaptive systems providing only the minimum support necessary to carry out the movement. This result is similar to the one observed by Krebs et al. (60). In fact, as Lynch et al. have shown, one needs to be careful to select the appropriate robot controller and adaptive settings otherwise assistance may not be the treatment modality optimizing motor recovery (25, 52). This is not limited to upper extremity; studies of robot-assisted gait training have demonstrated that such rehabilitation is more effective when the user actively participates in the movement. Passive guidance has a null impact on recovery in stroke patients (61).

Regarding treatment modalities, Krebs et al. (62) showed that a robotic device did not confer any benefit when the transport phase of the arm (approach phase) was combined with the taking and handling of real objects (functional rehabilitative approach) compared with repeated movements whose sole goal was to reach virtual targets (single phase approach). A similar result was obtained by Milot and colleagues. They employed the same robot and compared functional training of whole arm vs. single phase approach and contrary to their hypothesis, found out that “breaking it down is better” (63).

The effect of the rehabilitation practice in bimanual movements was also assessed with a robotic device that enables this type of work. Hesse et al. (64), and Lewis and Perreault (65) have suggested improved coordination after a rehabilitation program in robot-assisted bimanual movements (vs. unilateral). However, Lum et al. (66) showed that contrary to his hypothesis, the bimanual mode led to lower improvements (close to the results of the control group) than those of the unilateral practice. These data corroborated the findings of the meta-analysis of Coupar et al. (67) on the lack of superiority of bimanual therapies.

## Rehabilitation Robot, a Tool to Alter Experimental Conditions

Robotic therapy enables us to properly control the experiment paradigm and assess the impact of environmental conditions on motor performance after a stroke. Thus, Krebs et al. (68) suggested that upper limb motor recovery would be impacted by an order-effect of treated limb segments. These authors utilized 2 different modules of the InMotion™ robots (commercial version of the MIT Manus robots), one for the proximal part of the upper extremity (shoulder/elbow module) and the other one for the wrist, and demonstrated that training the more distal segment first led to greater skill transfer to the proximal limb segment than vice-versa. The issue of the influence of exercise practice on brain plasticity depending on the treated joint (proximal or distal) had also been studied in neurophysiology (69) but was only slightly taken into account in rehabilitation protocols. However, it has been suggested that use-dependent plasticity would have a distal-to-proximal gradation with likely consequences on brain remapping and implications in rehabilitation regimens. Krebs et al. (68) also described the contribution of robotic devices to the exploration of temporal interference occurring within motor learning process. Previously, Brashers-Krug et al. (70) suggested that the consolidation of a motor skill, a sequence of the motor learning process, was disrupted when a second motor task was learned immediately after the first due to a potential phenomenon of motor interference, but not if the second task was learned at least 4 h later (70). Conroy et al. (71) studied the effects of performing 2 distinct motor tasks using 2 different shoulder/elbow robots of the InMotion™ series within the same training session; one emphasizing planar gravity compensated reaching movements and the other one spatial reaching movements (without gravity compensation). The results suggested a potential temporal interference of the first task on the other one, finding a mild superiority of the protocol using only one task (planar gravity compensated robot) compared to the protocol combining both tasks, i.e., reaching in a gravity compensated space and movement against gravity (spatial task).

The effects on motor learning of the type of feedback provided to patients during practice have also been addressed using robotic devices. Robotic tools offer patients various forms of feedback (visual, auditory, haptic…) and provide patients with different forms of knowledge of results (how many successes) or of their motor performance (number of repetitions, amount of assistance, deviation from straight lines…). A growing body of results demonstrated that this feedback information can not only optimize patient's motivation and engagement but can also enhance learning and recovery (72–74). While most researchers employ different forms of visual feedback, Rosati et al. (75) and Secoli et al. (76) demonstrated that motor performance could be also enhanced when an auditory feedback was part of robot-assisted training in stroke survivors.

## Robots as Objective Evaluation Tool Affording Insights Into Motor Recovery Process

An important aspect that must be highlighted is that rehabilitation robots are remarkably good evaluation tools, allowing an accurate characterization and quantification of time-course evolution of motor performance. Most advanced robotic systems include sensors which measure and record kinematic and kinetics during upper extremity movement used to derive indicators and movement features. For example, Krebs et al. (77) analyzed kinematic characteristics of movements performed by 20 patients within the period of motor recovery after stroke and found that the purportedly continuous hand trajectory was actually segmented into apparent submovements. Further experiments showed that in the early stages of recovery, there is minimal overlap of sub-movements. As recovery proceeds, movement initially becomes less smooth and then smoother (78, 79). Rohrer et al. (80) attributed this result to a progressive overlapping and blending of submovements.

Pila et al. (45) found that a 3-month intensive rehabilitation program was associated with improvement in shoulder and elbow movements first, which suggests focal behavior-related brain plasticity. From this study, results also suggested that improvement of movement quantity related parameters might precede that of movement quality (accuracy). In a previous study, using a novel approach by analyzing interaction parameters, Duret et al. (81) found that movement velocity recovered before accuracy and that the negative correlation between lateral guidance and the velocity-related parameter shown in this study might suggest a speed/accuracy tradeoff in motor performance.

Duret et al. (82) and Subramanian et al. (83) further demonstrated that kinematic indicators might be valid measures for assessing upper limb motor impairments and suggested that these data would complement clinical assessment. Duret et al. (82) and Zollo et al. (84) showed that kinematic measures were only moderately correlated with Fugl-Meyer scores, particularly with the proximal part of the FMA upper extremity scores (83). Moreover, several studies (82, 85, 86) found that kinematic measures of the hand trajectories were responsive measures for capturing improvements in the upper extremity during the first months after stroke. Mirbagheri and Rymer (87) suggested that kinematic measurements such as the active range of motion (AROM) might be a reliable indicator of motor recovery. They suggested that the Fugl-Meyer score (88) at 1 year post-stroke could be predicted from the AROM measurement at 1-month. Bosecker et al. (89) demonstrated that robotic measurements could adequately predict the clinical scales. Krebs et al. (90) expanded this result beyond Bosecker's linear correlation and employing non-linear methods, he obtained a much better correlation. In fact, he demonstrated a much improved effect-size with the robotic-assay suggesting a reduction of 70% in patient's census, establishing a new biomarker to assess new pharmacological agents.

## Robotics Within the Rehabilitation Process

Our results and those of others suggest that robotics can be integrated in clinical practice. It is our opinion that training should consist of a series of robot-training sessions interspaced by sessions in which the clinicians assist patients to translate their impairment gains into function. For example, Hung and colleagues (18) trained 21 persons with chronic stroke for 20 sessions (5 times per week, for 4 weeks) with the MIT-wrist robot followed by transition-to-task training or with the wrist robot followed by impairment-oriented training (same total time in therapy). Both groups improved significantly, but they observed a change of 8.1 and 2.7 points in the Fugl-Meyer scale, respectively. Their results highlight the boosting effect of the transition-to-task sessions. Their finding is in line with our long standing view that in a clinical setting, robotic therapy should focus on impairment with the therapist tailoring therapy to the particular patient's need and assisting in translating impairment gains into function. Of course our proposed two-step approach of robotics followed by transition-to-task represents our working model, and as such, it will continue to evolve as more evidence is gathered.

## Rehabilitation Robotics: Toward Widespread Access

One of the biggest limitations on the widespread access of the technology is its cost. Presently rehabilitation robotic devices are priced in the range of $75,000–$350,000 US dollars prior to any additional hidden costs related to shipping, taxes, maintenance, and installation/training. This is a particularly ominous limitation as 85% of all stroke deaths occur in low and middle-income countries (LMIC). Yet, the greatest burden caused by stroke is not related to death as three-fourths of stroke victims survive the acute injury. Long-term impairment, limitation of activities (disability), and reduced participation (handicap) have dramatic consequences for individuals, families and societies. The burden from stroke is expected to increase over the next decades in LMIC, even with improvement in preventive measures and better acute care, due to global graying of the population-since stroke incidence increases with age (91). Therefore, efforts to decrease disability from stroke must parallel measures to prevent stroke; they must be realistically adapted to conditions of LMIC and not limited to high income countries HIC. On the positive side, there is an expectation that clinic based rehabilitation robots will ultimately cost as much as “popular” car models. If so, in an era of constraint budget associated with decreasing length of hospital stays worldwide, robotics has the potential to increase the productivity and quality of care after stroke in all countries.

## Limitations

One needs to take this overview with the appropriate caveats. The scope of work was limited by the methodology employed: this overview was not a rigorous systematic review or meta-analysis. Instead it reflected the authors opinions and working model incorporating an aggregated over a total of 50 combined years of practical observations upper extremity robotic training in clinical and research settings.

## Conclusion

Robotic therapy has matured and represents an embodiment of a paradigm shift in neurorehabilitation following a stroke: instead of focusing on compensation, it affords focus in ameliorating the impaired limb in line with concepts of neuroplasticity. This technology-based treatment provides intensity, interactivity, flexibility, and adaptiveness to patient's performance and needs. Furthermore, it increases the productivity of rehabilitation care. Of course, efficiency must be discussed within a local perspective. For example, following the cost containment shown in the VA ROBOTICS study (46), the UK National Health Service commissioned the RATULS study to assess costs within the British system (29). As we expect the RATULS study with 770 stroke patients to be published in June 2019, one might consider how the technology might affect local costs of deployment and expand robotic therapy beyond the clinic to the community and home.

## Author Contributions

CD and HK chose the article's subject, wrote and reviewed the manuscript. A-GG checked the reference list, reviewed the manuscript and formatted the manuscript.

### Conflict of Interest Statement

HK is a co-inventor in MIT-held patents for rehabilitation robotic devices. He was the founder of Interactive Motion Technologies and former Chairman of the Board of Directors (1998–2016). He founded 4Motion Robotics (2017). The remaining authors declare that the research was conducted in the absence of any commercial or financial relationships that could be construed as a potential conflict of interest.
